# FRAILTY ASSESSMENT BASED ON THE QUALITY OF DAILY WALKING

**DOI:** 10.1093/geroni/igz038.328

**Published:** 2019-11-08

**Authors:** Danya Pradeep Kumar, Nima Toosizadeh, Jane Mohler, Kaveh Laksari

**Affiliations:** Biomedical Engineering, University of Arizona, Tucson, Arizona, United States

## Abstract

Frailty is an increasingly recognized geriatric syndrome resulting in age-related decline in reserve across multiple physiologic systems. An impaired physical function is a prime indicator of frailty. In this study, we aim to implement a body-worn sensor to characterize the quantity and quality of everyday walking, and establish associations between gait impairment and frailty. Daily physical activity was acquired for 48 hours from 125 older adults (≥65 years; 44 non-frail, 60 pre-frail, and 21 frail based on the Fried gold standard) using a tri-axial accelerometer motion-sensor. Continuous purposeful walks (≥60s) without pauses were identified from time-domain acceleration data. Power spectral density (PSD) analysis was performed to define higher gait variability, which was identified by a shorter and wider PSD peak. Association between frailty and gait parameters was assessed using multivariable nominal logistic models with frailty as the dependent variable, and demographic parameters along with the gait parameters as the independent variables. Stride times, PSD gait variability, and total and maximum continuous purposeful walking duration were significantly different between non-frail and pre-frail/frail groups (p<0.05). Using a step-wise model with the above qualitative and quantitative gait parameters as predictors, the pre-frail/frail group (vs. non-frail) was identified with 71.4% sensitivity and 75.4% specificity. Everyday walking characteristics were found to be accurate determinants of frailty. Along with quantitative measures of physical activity, qualitative measures are critical elements representing the stages of frailty. In-home gait analysis is advantageous over clinical gait analysis as it enables cost- and space-effective continuous monitoring.

